# Combination Cancer Therapy and Reference Models for Assessing Drug Synergy in Glioblastoma

**DOI:** 10.3390/curroncol33010019

**Published:** 2025-12-29

**Authors:** Semyon A. Sinyavskiy, Nelly S. Chmelyuk, Daria Yu. Travnikova, Vsevolod V. Belousov, Tatiana O. Abakumova

**Affiliations:** 1 Department of Synthetic Neurotechnologies, Pirogov Russian National Research Medical University, Ministry of Health of the Russian Federation, Moscow 117513, Russiatravnikova_diu@rsmu.ru (D.Y.T.); belousov_vv@rsmu.ru (V.V.B.); abakumova_to@rsmu.ru (T.O.A.); 2 Federal Center of Brain Research and Neurotechnologies, Federal Medical Biological Agency, Moscow 117513, Russia

**Keywords:** glioblastoma, anticancer therapy, combination therapy, drug synergy, combination index

## Abstract

Glioblastoma is currently an incurable disease despite the development of a wide variety of therapeutic approaches, from surgical methods to immunotherapy. In current clinical practice, treatment typically involves a combination of existing methods, often comprising three stages: tumor resection, radiotherapy, and chemotherapy. Modern research offers improved chemotherapy strategies, as well as combinations of chemotherapy with immunotherapy. However, the efficacy of these therapies is profoundly influenced by factors such as tumor and peritumoral heterogeneity, alongside complex molecular signaling pathways. Optimizing glioma treatment requires a rigorous mechanistic understanding of individual approaches and their synergistic effects. This review comprehensively details current glioblastoma therapeutic strategies and critically evaluates key reference models for assessing combination therapy efficacy and their inherent limitations. A deeper understanding of these mechanisms and models will refine the investigation of observed therapeutic effects and accelerate the translation of promising *in vitro* approaches to effective clinical management of malignant gliomas.

## Simple Summary

This review provides an overview of current and emerging glioblastoma treatment approaches, with particular attention to combination therapies and the reference models used to assess their effectiveness. Identifying true synergistic effects between therapeutic agents is critical for improving patient outcomes. Properly designed reference models that reflect the mechanisms of action across preclinical and clinical stages can significantly enhance the interpretation of experimental data. A deeper mechanistic understanding and more accurate modeling will ultimately accelerate the translation of promising combination therapies into effective clinical strategies for managing malignant gliomas.

## 1. Introduction

Glioblastoma is a heterogeneous tumor characterized by a high degree of malignancy and poor clinical outcomes. On average, patient survival ranges from 14 to 16 months following diagnosis, even when standard therapeutic protocols are applied [1]. These conventional regimens typically do not account for the molecular and biomarker profiles of tumor cells, which significantly limits the ability to personalize treatment. Due to the genetic heterogeneity of glioma cells, the disease remains highly resistant to therapy, and relapse is common following treatment [2]. Consequently, the need to modernize current therapeutic strategies remains urgent. The standard glioblastoma treatment protocol generally consists of several stages. The first stage involves surgical resection, which aims to mechanically remove tumor tissue. However, this approach carries a substantial risk of damage to surrounding healthy brain tissue, and complete eradication of all malignant cells is virtually impossible due to the invasive, infiltrative nature of glioblastoma growth; as a result, recurrence is almost inevitable [3]. Another conservative approach of glioma treatment is radiotherapy, which employs focused ionizing radiation to target tumor tissue. Although effective, this method requires complex patient preparation and is associated with multiple side effects. The success of treatment depends heavily on tumor radiosensitivity; as with surgical resection, recurrence rates remain high [4]. Chemotherapy represents an integral part of glioblastoma management, which typically involves the administration of small-molecule drugs with broad effects on tumor cell processes, such as temozolomide (TMZ), vincristine, procarbazine, and others [5]. However, these agents often lack target specificity, resulting in high systemic toxicity and collateral damage to healthy cells and tissues [6,7]. Furthermore, glioma cells frequently exhibit compensatory mechanisms that attenuate the cytotoxic effects of chemotherapeutic agents, leading to therapeutic resistance [8,9]. Immunotherapy has emerged as one of the most promising areas for glioma treatment. It encompasses a range of modalities, including chimeric antigen receptor (CAR)-T cell therapy, immune checkpoint inhibition, therapeutic vaccines, and oncolytic virotherapy [10]. Nonetheless, the efficacy of immunotherapy in gliomas is limited by multiple factors—most notably, the pronounced cellular heterogeneity of the tumor, the presence of an immunosuppressive microenvironment, and the restricted permeability of the blood–brain barrier (BBB). Despite the rapid development of chemo- and immunotherapeutic approaches, treatment efficacy in gliomas remains markedly insufficient. Several innovative strategies have been developed in recent years, yet most have demonstrated limited success as monotherapies and have failed to show significant benefit in phase III clinical trials [11,12].

Based on the above, it can be concluded that no single therapeutic modality, when applied in isolation, can achieve a substantial improvement in patient survival. Chemotherapeutic agents, while clinically established, offer limited efficacy, exhibit high toxicity, and negatively affect patients’ quality of life.

In current clinical practice, combination therapies are increasingly employed—for example, radiotherapy followed by chemotherapy, often involving multiple chemotherapeutic agents [13]. The development of novel treatment strategies for glioblastoma remains a central challenge in oncology, requiring a reevaluation of existing therapeutic paradigms and the exploration of rational drug and modality combinations. However, a key difficulty lies in accurately assessing the efficacy of combination treatments, as determining the synergistic or antagonistic nature of drug interactions remains complex. Numerous evaluation methods and reference models have been proposed, yet selecting the most appropriate approach for a given context is often unclear. Another persistent challenge is translating successful *in vitro* combinations into *in vivo* and clinical settings, as well as integrating these findings into new treatment protocols. Despite these challenges, a growing number of preclinical and clinical studies have demonstrated the potential of combination therapies employing diverse pharmacological agents and molecular targets.

The objective of this review is to examine the prospects for using novel agents with synergistic properties to enhance the efficacy of first-line therapies in patients with glioblastoma, as well as to discuss existing mathematical models used to quantify and predict synergistic effects.

## 2. Current Clinical Drug Combinations in Glioblastoma

TMZ is the basis chemotherapy for glioblastoma [14]. For nearly two decades, TMZ has been used alongside surgery and radiotherapy as part of the standard treatment regimen according to the Stupp protocol. Its advantages include a relatively favorable safety profile and an improvement in overall survival among patients with newly diagnosed glioblastoma, which has led to its establishment as the worldwide standard of care in chemotherapy [15,16]. It is converted to an active metabolite that alkylates guanine at the O-6 and N-7 positions, contributing to its antitumor activity in cells [14]. TMZ exhibits high oral bioavailability and efficient penetration in the nervous system due to its low molecular weight and lipophilicity. However, therapeutic efficacy is strongly modulated by tumor-intrinsic molecular characteristics. Initial sensitivity to the drug is linked to the methylation of the O-6-methylguanine-DNA methyltransferase (MGMT) gene promoter [17] and the presence of mutations in isocitrate dehydrogenase (IDH) gene [18,19]. As a result, more than half of patients possess primary or acquired resistance to TMZ, limiting its usage as monotherapy [20]. To enhance therapeutic efficacy and overcome resistance, it is preferable to use combination regimens rather than single-agent therapy. For instance, current treatment plans include different drugs to address various resistance mechanisms. Clinical regimens could be separated into four different groups according to their mechanism of action:

(1) Increasing the intensity of treatment within one pathway (parallel or dual inhibition): this regimen requires concurrent administration of two or more agents that target the same signaling pathway or molecular process could result in additive or synergistic effects and reduce compensatory feedback.
(2) Increasing the intensity of treatment by targeting separate cellular pathways: this approach reduces tumor resistance by simultaneously disrupting different vital cell functions or pathways.
(3) Integration of chemotherapy with immunotherapy: chemotherapy may increase tumor antigen release and modulate the tumor microenvironment, potentially improving immune recognition and effector function.
(4) Integration of chemotherapy with drugs against specific mutations: combining nonspecific cytotoxic agents with targeted therapies against tumor-specific genetic alterations could provide broad cytotoxic pressure.

As demonstrated in Table 1, there are ten currently clinically available protocols with drug combination recommended by Russian Society of Clinical Oncology (RUSSCO) [21]. We selected RUSSCO guidelines because they explicitly present multiple combination therapy variants that are directly relevant to the treatment strategies discussed in our manuscript. International guidelines from American Society of Clinical Oncology (ASCO) and The Society for Neuro Oncology (SNO) or National Comprehensive Cancer Network (NCCN) overlap in many areas but differ in emphasis. ASCO–SNO presents fewer definitive recommendations for recurrent glioblastoma, while NCCN provides performance-status-based options and includes both single-agent and combination approaches [22,23,24,25]. Given our focus on combination regimens, RUSSCO offered the most relevant and unambiguous source. For instance, a combination regimen with lomustine, vincristine, and procarbazine was first implemented for the treatment of recurrent glioblastoma in 1975 [26]. It was proposed that combining these drugs might extend survival because each drug blocks tumor growth by a different mechanism. Lomustine, a nitrosourea alkylating drug, damages DNA and RNA through the formation of O-6-chloroethylguanine adducts, resulting in the formation of strand cross-links and subsequent cell death [27,28]. Vincristine, a naturally occurring plant alkaloid, interacts with β-tubulin, thereby preventing the function of microtubules during the metaphase of mitosis. However, it does not adequately penetrate the BBB; its efficacy might therefore be attributed to partial BBB disruption observed in grade 3–4 gliomas [29,30,31]. Procarbazine is another alkylating agent, but it is less cytotoxic compared to TMZ [32,33]. Moreover, this drug additionally methylates transport RNA, which reduces protein biosynthesis and indirectly influences reactive oxygen species (ROS) production.

The NCT00052455 trial comparing procarbazine–lomustine–vincristine versus two TMZ regimens (five-day and three-week regimens) was conducted in the recurrent glioblastoma; enrolled patients had received prior radiotherapy but did not undergo chemotherapy and were enrolled in the study within two weeks of first MRI-documented recurrence [34]. It was shown that the nine-month survival rate was 17% in patients receiving combination therapy, 26% in the TMZ-5 group, and 13% in the TMZ-21 group. The incidence of acute toxicity was comparable in all three groups. Within the TMZ groups, the 21-day regimen demonstrated less favorable outcomes in comparison to the conventional 5-day regimen, which does not support the idea of increasing dose of TMZ through longer administration.

It should be mentioned that the majority of combination therapy trials, excluding modifications of TMZ protocol, are conducted in patients with recurrence rather than at initial diagnosis. Recurrence narrows the window for effective intervention: tumors at this stage frequently display more aggressive biology, treatment resistance, and reduced performance status in patients, all of which limit therapeutic options and trial eligibility [35,36,37].

An alternative strategy focuses on enhancing the effect of drugs that act on the same molecular pathway or target. Examples of such combinations include procarbazine with lomustine, or TMZ with cisplatin or carboplatin. All of these agents are alkylating cytostatics, whose primary mechanism involves the addition of an alkyl group to DNA [38]. This, in turn, disrupts DNA, prevents mitosis and triggers apoptotic pathways in the damaged cell [39].

Despite lacking reactive alkyl groups, carboplatin and cisplatin induce DNA damage via a mechanism functionally analogous to alkylating agents [40]. These platinum-based compounds form stable DNA-platinum adducts, which subsequently mediate the formation of intra- and interstrand DNA cross-links. The resulting structural modification impedes replication and transcription machinery, triggering DNA damage response signaling [41].

Nevertheless, the main challenge in treating by alkylating agents is high MGMT activity. It was demonstrated that cisplatin is able to reduce MGMT activity in a time-and dose-dependent manner *in vitro*, which in turn may enhance therapeutic efficacy [42]. This effect is supported by increased cytotoxicity in several solid tumor models treated with cisplatin combined with TMZ at subtherapeutic concentrations [43].

A comparative analysis of three clinical phase II trials assesses the efficacy of TMZ combined with cisplatin versus TMZ as monotherapy varying dosing schedules and regimens [31,44,45]. Combination therapy demonstrated a clinically meaningful advantage: 6-month progression-free survival was 34%, and 6-month overall survival reached 81%, meanwhile the best corresponding outcomes in TMZ cohorts were 24% and 64%, respectively [46]. These findings suggest that adding cisplatin to TMZ may improve short-term disease outcomes and survival, although cross-trial comparisons should be interpreted cautiously due to potential differences in patient populations, dosing, and study design [32,44,45,46]. At the same time, this combination proved to be ineffective for stage III–IV gliomas in children [47]. To overcome resistance in pediatric and adolescent patients, it may be necessary to target additional resistance mechanisms—for example, deficiencies in base excision repair.

The third therapeutic strategy for gliomas involves combining bevacizumab with one of three approved chemotherapeutic agents: TMZ, irinotecan, or etoposide. Bevacizumab is a monoclonal antibody that selectively binds to and neutralizes vascular endothelial growth factor (VEGF), thereby inhibiting VEGF-driven signaling [48]. Its mechanism centers on anti-angiogenesis: by suppressing the formation of new blood vessels, the tumor’s access to oxygen and nutrients is progressively decreased [49]. As a result, pro-angiogenic signals are blocked, slowing the aggressive progression of the neoplasm and creating a microenvironment more amenable to chemotherapeutic agents [50].

Bevacizumab is used in two regimens with topoisomerase inhibitors. Etoposide, a topoisomerase II inhibitor, induces double-strand DNA breaks in rapidly dividing cells and acts predominantly during the S phase of the cell cycle, with additional activity in G2 at higher doses [51,52]. Cytotoxic effects on normal healthy cells are generally observed only at high dose levels [53]. Irinotecan acts through stabilization of the complex formed between topoisomerase I and DNA, preventing ligation of single-strand breaks [53,54]. Synergy between bevacizumab and topoisomerase inhibitors is achieved through complementary effects on the tumor microenvironment and tumor metabolism, where angiogenic remodeling and impaired vascular supply sensitize tumor cells to DNA-damaging agents and may improve drug delivery and efficacy [55].

Bevacizumab induces normalization of tumor vascularization and reduces abnormal permeability [56]. By improving vascular structure and function, this remodeling enhances intratumoral delivery of cytotoxic agents, increasing their local concentration and potentiating antitumor activity [55]. In the absence of such effects, tumors with chaotic, immature vasculature often exhibit regions of hypoperfusion and barrier-like permeability, which limits drug penetration into deeper tumor compartments [57].

The last combinational treatment approved for clinical use is based on specific gene alteration inhibition, such as BRAF and MEK inhibitor combinations, for example, dabrafenib plus trametinib or vemurafenib plus cobimetinib. These regimens have shown substantial therapeutic promise for gliomas with activated BRAF mutations, which occur in approximately 1–2% of adult patients [58]. Dabrafenib is a selective BRAF kinase inhibitor that targets the constitutively active mutant BRAF protein (commonly BRAF V600E), a critical driver of the RAF/MEK/ERK signaling cascade [59,60]. In tumors bearing the BRAF V600E mutation, BRAF is locked in an active state that leads to uncontrolled cell proliferation and oncogenic signaling [61]. When given in combination with MEK inhibitors, BRAF-targeted therapy produces more durable responses and mitigates mechanisms of resistance that arise from reactivation of downstream signaling. Although the prevalence of BRAF mutations in adult gliomas is low, identifying these alterations clinically important because affected patients may possess meaningful benefit from targeted therapy [58,59,60,61]. Trametinib complements dabrafenib as a MEK inhibitor—targeting the kinase immediately downstream of RAF in the RAF/MEK/ERK signaling cascade [62]. MEK phosphorylates and activates ERK, thereby amplifying oncogenic signaling. Inhibition of BRAF by dabrafenib suppresses the upstream activation step of the RAF/MEK/ERK pathway, while trametinib blocks MEK-dependent ERK activation and limits bypass mechanisms that could restore pathway output despite BRAF inhibition [63]. Together, this dual blockade more effectively interrupts signal transmission to the nucleus, downregulates expression of genes that drive tumor proliferation, and promotes apoptotic programs in cancer cells [64].

Another example of targeted treatment for GBM is vemurafenib and cobimetinib. The first agent represents another prominent BRAF inhibitor, specifically designed to target the V600E/K mutations [65,66]. This critical blockade disrupts the entire RAF/MEK/ERK signaling cascade, ultimately leading to a significant reduction in ERK phosphorylation and the suppression of oncogene transcription. In parallel, cobimetinib acts as a MEK1/2 inhibitor, functionally similar to trametinib but with higher selectivity [67,68].

In clinical practice, combination therapy for gliomas is founded on the principle of pharmacologic synergy: co-administration of agents can produce a greater therapeutic effect than monotherapy. This synergy arises from simultaneous targeting of multiple tumor supporting processes—for example, cell-cycle progression, DNA repair pathways, angiogenesis, and mechanisms of drug resistance—thereby increasing the probability of interrupting the complex biology that sustains tumor growth. Nonetheless, clinical data indicates a more complex and intricate reality. It was reported combinations do not clearly outperform monotherapy or provide benefits only in certain patient subgroups [46,47]. These observations underscore the need for deeper understanding of the molecular basis of synergistic interactions and the search for new, rational drug combinations based on the biochemical characteristics of tumor metabolism and resistance. Moreover, the findings highlight the difficulty of assessing true pharmacological interactions between therapeutics and issues in *in vitro* research to *in vivo* studies and clinical implementation.

## 3. Current Targets and Strategies in Drug Combination Therapy and the Biochemical Principles of Synergy

Despite the various approaches to combination therapy currently applied in clinical practice, the search for new targets whose modulation would exert a synergistic effect with standard-of-care drugs remains a serious challenge. Identification of such targets may reduce the emergence of drug resistance in gliomas and enhance therapeutic efficacy by concurrently targeting multiple molecular pathways. Ultimately, this could improve patient survival and/or quality of life. Moreover, the synergistic action of several drugs is expected to decrease overall chemotherapy-associated toxicity by allowing the use of lower effective doses while maintaining the desired therapeutic response. In addition, combination therapy has the potential to affect heterogeneous tumors, including stem cell populations, which positively influences the likelihood of achieving remission [69].

New approaches to target discovery are increasingly based on exploiting synergistic effects mediated through multiple molecular sites of action—that is, different compounds act on specific oncogenic signaling pathways characteristic of gliomas, such as RTK/PI3K/AKT/mTOR, RAS/RAF/MEK/ERK, and others, which are often overexpressed compared to normal tissues [13]. Prominent examples include combinations of receptor tyrosine kinase (RTK) inhibitors (e.g., EGFR, PDGFR) with mTOR or MEK inhibitors. Such combinations demonstrate stronger suppression of cell proliferation and survival than monotherapy. Specific cases are discussed in detail below.

Among the key oncogenic drivers, epidermal growth factor receptor (EGFR) regulates both the PI3K/AKT/mTOR and Ras/Raf/ERK pathways, whose activation contributes to tumor progression [70]. Consequently, EGFR represents a promising therapeutic target, particularly in tumors harboring amplification of the constitutively active variant EGFRvIII [71]. Pharmacological inhibition of this target has demonstrated partial clinical success; however, prolonged exposure often results in derepression of biochemical cascades in mouse astrocytes and in the human glioma cell line U87 [72,73,74]. Subsequent studies revealed that, alongside EGFRvIII, cross-activation of cMET RTK–associated signaling pathways—including PI3K/AKT, Ras/MAPK, JAK/STAT, SRC, and Wnt/β-catenin, among others—occurs in parallel [8]. This compensatory signaling maintains glioma cell migration and growth even under EGFRvIII inhibition [75], thereby diminishing therapeutic efficacy. Notably, concurrent blockade of multiple receptor pathways using erlotinib, a first-generation EGFR inhibitor, in combination with a cMET inhibitor, produced substantially greater tumor growth suppression compared with erlotinib monotherapy [76].

As noted above, a distinctive feature of RTK signaling in gliomas is their ability to compensate for blockade of these receptors-bounded signaling pathways through feedback mechanisms, reactivating suppressed cascades or initiating alternative, yet functionally similar, signaling routes [9,77]. Consequently, the use of drug combinations targeting parallel cascades in glioma cells is more effective than single-agent therapy. For instance, afatinib can concurrently suppress the EGFRvIII–cMET crosstalk in glioma stem cells. It has been shown that the combination of afatinib with TMZ reduces cancer cell proliferation by suppressing the expression of Oct3/4 and Nanog *in vitro*. The *in vivo* experiments showed the U87EGFRvIII glioma xenograft model exhibited slower progression upon combined administration of these agents [76]. In contrast, neither drug alone demonstrated comparable efficacy, indicating a synergistic interaction between the two treatments.

Another example is that inhibition of EGFR leads to rapid activation of an adaptive pathway driven by tumor necrosis factor (TNF) expression and initiation of the JNK–Axl–ERK cascade, which counteracts the desired cytotoxic effect of EGFR inhibitors [9]. Studies indicate that concurrent inhibition of EGFR and TNF holds therapeutic potential for glioblastomas lacking MGMT promoter methylation [78].

Targeting multiple nodes within a single pathway is also a promising approach to identifying compounds with synergistic effects. For example, AZD8055, a selective dual inhibitor of mTORC1 and mTORC2, was shown to induce autophagy *in vitro* and suppress tumor growth *in vivo* [79]. When combined with TMZ, this treatment regimen significantly increased animal survival in *in vivo* experiments [80].

In addition, interferons are known to suppress the NF-κB pathway, thereby sensitizing glioma stem cells to TMZ therapy via downregulation of MGMT expression [81]. Clinical trials have also been conducted in which patients with high-grade gliomas received combination therapy with TMZ and interferon-α. The study demonstrated that patients treated with the combination had a median overall survival of 26.7 months compared with 18.8 months in the TMZ monotherapy group. Thus, the combined administration of TMZ and interferon-α prolonged survival in glioblastoma patients regardless of MGMT promoter status, while maintaining an acceptable toxicity profile [82].

Another promising direction involves combinations that target general disruptions of cellular homeostasis, including redox balance. In addition to the glutathione system, which is responsible for antioxidant defense and maintenance of cellular homeostasis [83], cells also contain the thioredoxin reductase (TrxR) system. This system plays a key role in sustaining cellular redox balance, regulating cell growth and apoptosis, and participating in cytokine and chemokine signaling [84]. It has been observed that glioma cell lines exhibit elevated expression of the TrxR system, which may be attributed to the fact that glioma cells are more susceptible to oxidative stress due to enhanced metabolism, leading to increased cytoplasmic ROS levels. Auranofin, an inhibitor of thioredoxin reductase I, has recently been investigated as an antitumor agent because of its pronounced cytotoxicity toward tumors, mediated by the induction of oxidative stress [85,86]. However, the clinical application of auranofin is limited by its poor solubility in hydrophilic media, necessitating the development of improved delivery strategies—either by enhancing solubility or employing delivery platforms such as nanoparticles [87].

Moreover, to enhance the efficacy of first-line agents such as TMZ in gliomas, it is possible to employ compounds that induce post-translational modifications of tumor cell proteins, thereby restoring the therapeutic effectiveness of the primary drug. Recent studies have shown that poly(ADP-ribose) polymerases (PARPs) interact with the DNA repair protein MGMT, which plays a particularly important role in glioma cell lines. However, in many gliomas, the promoter of this gene is unmethylated, leading to active genomic repair following TMZ-induced alkylation. In this context, MGMT interacts with PARP, resulting in increased MGMT activity. The use of PARP inhibitors—including olaparib, niraparib, talazoparib, and others—prevents the PARylation, i.e., the attachment of poly(ADP-ribose) fragments to MGMT, thereby impairing DNA repair in glioma cells due to reduced removal of TMZ-induced methylated DNA products, eventually restoring tumor sensitivity to TMZ [17]. This combination could benefit patients with long-standing, recurrent tumors, which over time may lose MGMT promoter methylation and regain their initial level of genomic repair capacity.

**Figure 1:**
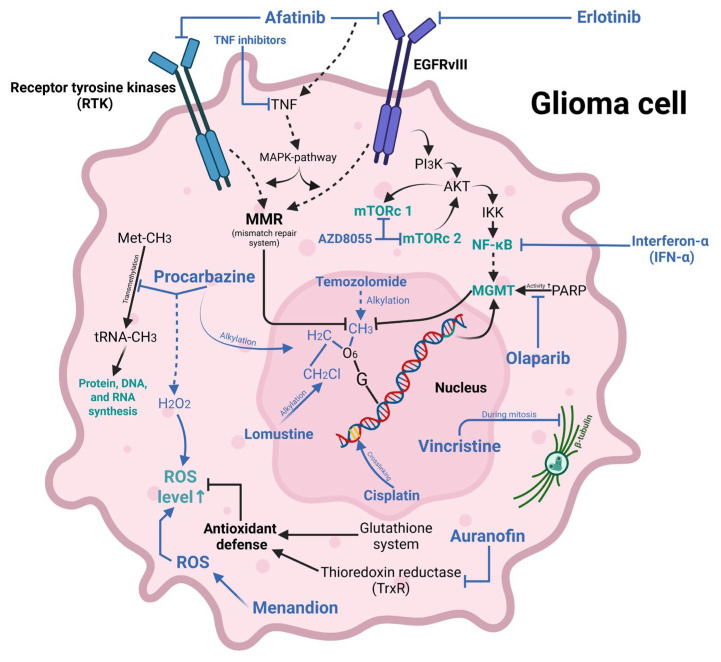
Effects of therapeutic agents on various biochemical processes in glioma cells.

In summary, numerous synergistic effects emerge when therapeutic agents target molecular pathways that exhibit mutual reinforcement—for instance, when a secondary pathway is activated as a compensatory mechanism upon inhibition of the primary one, or when drugs act on different targets within a single, branched signaling cascade. All of the discussed targets and drugs were schematically showed in Figure 1. This figure was created in BioRender, with access link: https://www.biorender.com/ (accessed on 28 October 2025). Researchers have long pursued such combinations, and emerging multi-class therapeutic strategies are now capable of eliciting diverse, including systemic, cellular, and tissue-level responses.

## 4. Novel Approaches in Glioblastoma Management

Despite significant progress in identifying potential drug targets for glioblastoma, clinical and experimental data on new treatment approaches are still being actively developed and require further testing to confirm their efficacy and safety. Drug combination studies are increasingly being used to find rational combinations that increase antitumor activity while reducing toxicity. A number of combinations are currently undergoing preclinical and early clinical testing.

Several additional points of therapeutic intervention deserve discussion as potential targets for combination strategies. One of the most promising directions involves the use of epigenetic modulators. For instance, vorinostat modifies chromatin architecture, resulting in the activation of tumor suppressor genes and the induction of apoptosis [88]. Similarly, azacitidine, a DNA methyltransferase inhibitor, promotes gene demethylation and restores normal gene function [89]. Another potential approach involves blockade of the immune checkpoint αCTLA-4, which induces an immune response through CD4^+^ T-cells and MHC-II+ microglial activation, enhancing phagocytosis and tumor clearance. However, targeting this pathway alone has shown limited efficacy, as other molecular mechanisms are thought to suppress an adequate antitumor immune response [90].

A rapidly advancing area of research is chimeric antigen receptor T-cell (CAR-T) therapy, which employs autologous T lymphocytes engineered to express chimeric receptors capable of recognizing tumor-associated antigens. This approach has achieved clinical success in the treatment of several hematologic malignancies, including lymphomas, B-cell leukemia, and multiple myeloma [91]. Among the promising targets for CAR-T therapy in glioblastoma is CD317 (Tetherin/BST2/HA1.24), whose targeting has been shown to suppress tumor growth and prolong survival in in situ and *in vivo* murine glioblastoma models [92]. Furthermore, a number of completed phase I–III clinical trials have evaluated CAR-T therapies directed against IL13Rα2, EGFRvIII, HER2, GD2, and EphA2 (NCT05241392, NCT04214392, NCT01454596, NCT02208362, NCT01109095), with several ongoing trials exploring an even broader spectrum of antigenic targets [92]. The principal selection criterion for these targets is high expression in glioma cells coupled with minimal expression in normal brain tissue, thereby maximizing tumor specificity while minimizing off-target effects. Despite its promise, CAR-T therapy in glioblastoma faces significant obstacles. Chief among these is the immunosuppressive tumor microenvironment, which impedes the infiltration and activity of immune effector cells. Moreover, increasing evidence indicates that gliomas can activate adaptive resistance mechanisms in response to immune pressure, further reducing the efficacy of this therapeutic modality [93].

Relatively recently another emerging therapeutic direction is oncolytic virotherapy, which utilizes natural or genetically modified viruses capable of selectively replicating within malignant cells and inducing oncolysis [94]. One of the most extensively studied examples is the herpes simplex virus (HSV), which can infect and lyse glioma cells. However, feedback activation of the IGF2BP3/MIB1/FTO signaling axis has been shown to induce NETosis on neutrophils, thereby suppressing viral replication and diminishing the overall cytolytic effect. To counter this, BET inhibitors, decreasing the expression of both IGF2BP3 and CSF3 in glioma cells, have been proposed as adjuvant agents to attenuate IGF2BP3-mediated NETosis, consequently enhancing viral replication and therapeutic efficacy [95].

A persistent challenge in glioma therapy is the restricted BBB permeability, which limits the delivery of many systemically administered agents due to their physicochemical properties. This has forced the development of novel methods for temporarily and locally disrupting the BBB, thereby improving drug delivery to the central nervous system. One of the most promising techniques involves focused ultrasound (FUS), which enables transient, localized BBB opening and significantly enhances drug bioavailability in brain tissue [96]. Preclinical *in vivo* studies have demonstrated that the combined use of chemotherapeutics such as doxorubicin with FUS-induced BBB disruption increases intracerebral drug concentrations while minimizing vascular injury associated with ultrasound exposure [97].

An extension of this approach involves sonosensitive or sonodynamic drugs, which selectively accumulate within tumor tissues and are activated by FUS exposure. Another ultrasound-based strategy, histotripsy, employs short, high-intensity acoustic pulses to induce cavitation, mechanically fragmenting tumor tissue without thermal damage to adjacent healthy structures [98].

Among the most promising developments in drug delivery systems is the use of nanoparticle-based platforms. Nanoparticles—engineered carriers with diameters typically below 100 nm—can adsorb or encapsulate a wide variety of therapeutic molecules, including chemotherapeutic agents and bioactive compounds [99]. Two major categories are distinguished: passively targeted nanoparticles, which exploit the enhanced permeability and retention (EPR) effect characteristic of tumor vasculature, and actively targeted nanoparticles, which employ physicochemical cues such as temperature, pH, or electric fields to achieve site-specific delivery [100]. The ongoing development of novel nanoparticle architectures aims to optimize pharmacokinetics and systemic clearance, thereby improving therapeutic efficacy while minimizing off-target toxicity. For instance, pH-sensitive nanoparticles conjugated to doxorubicin via acid-labile linkers have demonstrated the ability to cross the BBB, distribute relatively homogeneously within glioma tissue, and exert robust antitumor effects [101].

Furthermore, nanoparticles can serve as efficient delivery vehicles for small interfering RNAs (siRNAs)—non-coding RNAs that bind to complementary messenger RNA (mRNA) sequences, leading to post-transcriptional gene silencing and mRNA degradation [102]. This mechanism offers the possibility of selectively suppressing genes implicated in drug resistance. For example, siRNAs targeting the MGMT gene can downregulate its expression by up to 90%, thereby resensitizing glioma cells with normal MGMT synthesis to TMZ [103]. Similarly, siRNA-mediated suppression of EZH2 expression in glioma models *in vivo* has been shown to inhibit tumor proliferation and promote apoptosis [104].

Nevertheless, despite the broad range of therapeutic strategies—both as monotherapies and in various combinations—glioblastoma remains an almost incurable disease. One potential avenue to address this challenge lies in the rational combination of existing treatment modalities; however, it is crucial to rigorously evaluate the outcomes of such combinations to avoid scenarios in which multimodal therapy proves less effective than monotherapy.

## 5. Reference Models for Assessing the Effect of Synergy Between Drugs

The effects of two drug compounds are largely determined experimentally *in vitro* and *in vivo*. When combining two or more drugs, the main goal is to achieve a maximum positive interaction effect compared to each drug used individually [105]. In essence, to achieve more with less. To maximize the effectiveness of drug combinations, it is important to find a way to prove that the combination has more benefits than any of the individual drugs. To quantify drug interactions, the observed effect of the drug combinations is often compared with the expected effect if there were no interaction between them, as predicted by a reference model. Drug combinations are tested at various doses, and their effects are plotted on a two-dimensional dose–response matrix. This matrix is then compared with the expected response calculated using the reference model. Typically, a reference model is created based on the dose–response relationship for a single compound, which can be modeled using the Hill equation [106].The Hill equation is used to model the relationship between the dose a of compound A and the response of cells y, when the cells are treated with the compound. The formula for this relationship is:

(1) y=ymin+ymax−ymin1+aa50h

where $y_{min}$ and $y_{max}$—minimum and maximum responses, respectively, $a_{50}$—dose producing 50% response (IC50 or EC50), h—the Hill coefficient, which indicates the degree of interaction between targets and chemicals and may take any value. Equation (1) can be rewritten as follows:

(2) E=y−yminymax−ymin=11+aa50h

which normalized response in the range from 0 to 1. Then it can be rewrite this response equation as a simple linear equation:

(3) f=1−E1hE=aa50

which is the median effect equation proposed by Chou. This equation shows that the ratio of non-response *(1-E)* to response *E*, when cells are treated with a compound is linearly proportional to the dose a. Several reference models have been proposed for single-compound dose–response modeling. The most common of these are the highest single agent (HSA), the Loewe additivity model, Bliss independence model, and zero interaction potency (ZIP) [106]. These will be briefly discussed below.

One important parameter is additivity, and it is not always obvious as it seems at first glance. First, let us review typical reference models that are most frequently cited and used in recent decades, and also touch on their limitations and applicability. All models can be divided into two main groups: (1) effect-based and (2) dose-effect approaches [107].

### 5.1. Effect-Based Approaches

These methods are based on the individual effects of each drug in a combination to estimate their interaction. There are four main strategies: combination subthresholding, HSA, response additivity and Bliss independent model. The term “effect” typically refers to cell viability or cell death, as it is a measurable response.

Let us introduce the following notation in the text: there are two drugs, *A* and *B*, used at concentrations *a* and *b* and with effects *E_A_* and *E_B_*. The effect of the drug combination is described by the expression *E_AB_*.

Consider the combination subthresholding: in this model, the subthreshold dos is the drug concentration that does not produce a significant effect compared to the negative control, i.e., ineffective in the conventional sense [108]. The combination subthresholding is the simplest approach, based on the idea that combining ineffective drug doses produces significant effects [109]. Unlike other reference models, this significant effect is determined by *p*-values obtained from statistical tests comparing the treated group to control group (untreated groups). The observed effect is considered statistically significant if the *p*-value < 0.05. However, the efficacy declared based on the threshold does not necessarily indicate a convincing difference between the drug combination effect and the individual drugs 96. Consider a case where the drug combination only just reaches statistical significance (for example, *p* = 0.049), and its individual components also just reach significance (*p* = 0.051). The reliability of the drug efficacy is ambiguous in this case, so no conclusion about synergy between them can be drawn. The combination subthreshold is sometimes used to study in detail the synergistic interactions of drugs or environmental influences. For example, the effect of the absence of cysteine and methionine on the induction of apoptosis in the presence of low doses of RSL3 was investigated in this study [109].

The next reference model is HSA (also known as Gaddum’s non-interaction model) [110]. In this model, a positive interaction occurs if the effect of the drug combination EAB is greater than the most potent individual drug. The combination index (CI) can be calculated using the following formula:

(4) $$CI=\frac{{max\;(E}_{A}{,\;E}_{B)}}{E_{AB}}$$

However, this model has limitations, and it is not always accurate. Therefore, it is important to consider other factors when making decisions about drug combinations. The significance of the positive effect of the combination is determined by the *p*-value of the statistical test that compares the combination with the single agent with the highest potency. HSA approach represents a slight improvement over the previous subthresholding combination approach, which compares meaningful effects rather than a combination effect versus the effect absence for individual agents [110]. In other words, this approach provides evidence of the superiority of the drug combination compared to individual agents. However, a positive outcome using the single-agent with the highest potency indicates a beneficial effect of the drug combination. Nevertheless, this provides limited evidence of synergy unless one of the drugs in the combination is known to be ineffective at any concentration [92]. Therefore, this model only considers the effect of a drug combination compared to the most potent individual agent, ignoring the potential additive effect of combining both drugs. This makes this model inappropriate for drug combinations where one drug is not effective at any concentration. This approach may lead to more optimistic results as it has a lower threshold for considering synergy compared to other models [107]. One typical example of using the HSA model is to study the sensitization of one drug to another, for example, the sensitization of TMZ with VAL-083 (Dianhydrodulcitol). In this case, the authors investigated the effectiveness of a given therapy used the HSA method (using SynergyFinder R software, detailed usage and installation are described by the authors early doi: 10.1007/978-1-4939-7493-1_17) to evaluate the efficacy of two drugs on glioma cell lines [111].

In addition, one commonly used model in practical studies is the additive response model. This model assumes that a positive interaction occurs when the combined effect of two drugs (*E_AB_*) is greater than the sum of their individual effects *(E_A_ + E_B_)*. The combination index can then be calculated using this formula:

(5) $$CI=\frac{E_{A}{+E}_{B}}{E_{AB}}$$

The appropriate *p*-value is determined by the significance of the interaction effect in a factorial analysis of variance, which takes into account both the individual and combined effects of the factors [112]. In comparison to the single-agent superiority model, the response additivity approach is more complex because it compares the observed combined effect (*E_AB_*) to the expected additivity effect, rather than the effects of the individual agents alone. This approach assumes that the drugs have linear dose–response curves with zero intercepts, but this is not always the case, as most dose–response curves have different shapes [105]. For example, if the two drugs have identical dose–response curves, the combined effect would simply be additive. Response additivity would indicate synergy in the upward-curved portion and antagonism in the downward-curve portion, leading to the misinterpretation that the combined effect of the drugs is less effective than the individual components. In practice, the same drug may exhibit synergy when used alone, but it is actually additive.

Another important method of calculation is the Bliss independence, which assumes that the drugs act independently on different targets. In this model, the effects of monotherapy and combined therapy are expressed as probabilities between 0 and 1 [107].

They can be compared to the expected additive effect, which is defined by the general formula for probabilistic independence *E_A_ + E_B_ − E_A_ = E_A_ + E_B_ − E_AB_*, where 0 *≤ E_A_ ≤* 1 and 0 ≤ *E_B_* ≤ 1. The resulting combination index can be calculated as:

(6) $$CI=\frac{E_{A}{+E}_{B}{-E}_{AB}}{E_{AB}}$$

Bliss independence is one of the most popular models for assessing drug combinations. However, it has several limitations. Firstly, the search for synergy involves drugs with complex, multiple, and unknown mechanisms of action. Secondly, the Bliss model assumes exponential dose–response curves for drugs, which can lead to misinterpretations similar to those for additivity. Finally, the model only applies to effects expressed as probabilities between 0 and 1 [107]. One application of Bliss’s independence is the use of TMZ and Niraparib, which act independently [113]. Each drug is acting independently (two drugs do not interfere with each other; the drugs have a different site/mechanism of action; and the drugs have exponential dose–effect curves).

### 5.2. Strategies Based on Dose–Response Curves

More modern approaches overcome the limitations of previously mentioned reference models. These methods are particularly useful for drugs with non-linear dose–response relationships, as they consider the amount of each drug needed to produce the same effect. In these approaches, the dose–response curves for each drug play a significant role, providing clear definitions for synergy, additivity, and antagonism. It is important to note that in order to determine the presence of an interaction between two drugs in a combination, it is necessary to first establish the non-interactive effects, which can be performed using null reference models.

#### 5.2.1. Loewe Additivity

The Loewe additivity model is a basic reference model and one of the most well-known approaches to dose–response. This model is based on the isobole representation to determine additive effects.

The Loewe method assumes that two drugs have similar mechanisms of action, but different potencies. In particular, the Hill coefficients for the two drugs must be identical. According to Equation (2), if two drugs *A* and *B* at different doses produce identical normalized responses (*E*), we have:

(7) E=11+aa50h=11+bb50h

or

(8) aa50=bb50

thus

(9) ba=b50a50=EAB50.

Equation (9) shows that the dose ratio of two drugs is independent of the response E and remains the same as the ratio of the IC50 or EC50 of the two drugs. It is worth noting that in the thought experiment, when the two drugs are identical, the effect of their action cannot be either synergistic or antagonistic. Next, consider the additive effect when using combinations of two drugs. We then need to define what “effects” are. If we are talking about cellular responses, such as survival, described by the Hill equation (Equation (2)), we cannot derive and show that the additive effects of two drugs are the sum of the individual responses due to the nonlinear dose-dependent term in the Hill equation. However, we can use one drug as an example to demonstrate our argument. For example, if we double the dose of drug *A* from a to 2*a*, we obtain the response *E*′:

(10) E′=11+2aa50h=11+2haa50h,

which is not equal to a twofold response when we treat cells with *a* dose E=11+aa50h due to the nonlinear term of the equation aa50. However, when considering effects as the ratio of non-response to response, these effects are linearly proportional to dose. In this case, we have $f\prime =\frac{2a}{a_{50}}$, which is essentially equal to 2f. Given this linear dose dependence, we are acting in accordance with Laplace’s insufficient principle, which states that if we have no or only limited information about the effects of two drugs in a mixture, when they interact with target proteins through the same/different mechanisms, the only logical conclusion regarding the additive effect of the two drugs is that it can only be considered as the sum of ratios $f_{A}$ for the drug *A* and $f_{B}$ for drug *B* with equal weighting factors. Namely,

(11) fad=fA+fB=aa50+bb50

Meanwhile, according to Equation (8), the modem will become:

(12) fef=aefa50=befb50=xx50

For response $E_{x}$, caused either by a mixture of doses of *x* or by individual drug *A* and *B* in doses *a* and *b*, respectively. Then it can be stated that $a_{50}=\frac{a_{ef}}{f_{ef}}$ and $b_{50}=b_{ef}/f_{ef}$. Substituting these values ($a_{50}$ and $b_{50}$) into in Equation (11) gives us:

(13) aaef+bbef=fadfef

If the value of $f_{ad}=f_{ef}$, then we have the Leow isobole, which states that the mixture effect equals the additive effect:

(14) aaef+bbef=1=CI

However, if the combination index (*CI*) is less than 1, it indicates a synergistic interaction, where the doses of the two drugs in the mixture are lower than expected based on additivity. If the *CI* is greater than 1, then there is antagonism, and the doses required for the combined effect are higher than predicted by additivity.

However, the isobologram method should be used with caution. It only works when the effect is defined as the ratio of the absence of a cellular response to a significant effect. The isobologram method can be applied to drugs with the same or different mechanisms of action. It is worth noting that this reference model assumes the principles of dose equivalence and dummy combinations. Dose *a* of drug *A* is equivalent to dose *b* of drug *B*, and dose *a* can be added to any dose of drug *B* to achieve an additive effect. This is not a characteristic of other reference models.

(15) $$ffect\left(a+b\right)=E_{A}\left(a+a_{b}\right)=E_{B}\left(b+b_{a}\right)=E_{AB}$$

Like previous reference models, the Loewe additivity model has some limitations. These limitations are primarily related to the dose–response curves of individual agents. The model is designed for drugs whose dose–response curves can be described by the Hill equation, also known as the sigmoid or logistic function. However, determining these curves can require a significant amount of data, making the process labor-intensive and time-consuming. Additionally, some drugs may have dose–response curves that are difficult to model, making the Leowe additivity model ineffective. Furthermore, many drugs do not follow a straight isobole, leading to non-parallel dose-response curves or when individual drugs do not achieve the same maximum effect [114].

Many null reference models are based on the isobole concept. The Zhou–Talalay approach, proposed by Zhou and Talalay, is still one of the most commonly used in biological research for quantitative assessment of drug interactions, especially synergism [106]. This reference model is based on the Leowe additivity model and includes the median effect equation, derived from the law of mass action principle of the unified theory. It is represented by the following equation:

(16) $$\frac{f_{a}}{f_{u}}={\left(\frac{D}{D_{m}}\right)}^{m}$$

where *D* is the drug dosage or concentration, fa is the fraction inhibited by drug dose *D*, $f_{u}$ is the unaffected fraction, $D_{m}$ is the dose causing 50% inhibition, and m is a coefficient indicating the shape of the dose–response curve. The model is also described by the value (*r*), which indicates whether the data conform to the law of mass action. The values of (*m*), ($D_{m}$), and (*r*) for each individual drug are the dose–response parameters required to implement the Zhou–Talalay theorem [115]. Thus, the unified theory establishes a general relationship between single and multiple entities, as well as first-order and higher-order dynamics. The general equation describing this previously mentioned model is derived from the Michaelis–Menten, Hill, Henderson–Hasselbalch, and Scatchard equations, which are most important in biochemistry and biophysics, and has also led to the development of the combination index theorem *CI*, which offers a quantitative definition of synergy in drug combinations [116]. The combination index for a combination of two drugs can be calculated as follows:

(17) $$CI=\frac{{\langle D\rangle }_{1}}{{\langle D_{x}\rangle }_{1}}+\frac{{\langle D\rangle }_{2}}{{\langle D_{x}\rangle }_{2}}$$

where ${\langle D_{x}\rangle }_{1}$ represents the dose of drug ${\langle D\rangle }_{1},$ which inhibits cell growth by x%, and ${\langle D_{x}\rangle }_{2}$ represents the dose of drug ${\langle D\rangle }_{2}$, which inhibits cell growth by x%. It has its own graphical representation, consisting of a plot of *CI* versus effect, where *CI* < 1, *CI* = 1, and *CI* > 1 indicate synergism, additivity, and antagonism, respectively. For example, the effect between 3-deazaneplanocin A, panobinostat, and TMZ was studied using this model via CompuSyn (version 1.0) software for calculating the combination index [117].

#### 5.2.2. Zero Interaction Efficiency (ZIP)

Zero Interaction Efficiency (ZIP) is a recent benchmark model proposed for estimating the expected responses of drug combinations. It is a hybrid approach that combines the Bliss independence model and the Loewe additivity. This model evaluates drug interactions by comparing the changes in the efficacy of dose–response curves between individual drugs and their combined effects [118]. The drugs are assumed to be independent of each other and do not interact when combined, leading to minimal changes in their response curves. This allows for accurate fitting of dose–response curves, which is crucial for determining parameters such as the concentration at half-maximal effect (EC50) and the slope. However, this approach can be challenging when dealing with low-quality data [119].

A major limitation in real-world experimental studies is that, in most cases, synergy is not assessed and calculated using appropriate reference models. Instead, the results are interpreted as synergy by comparing them with the results from simple experimental assays. This approach does not incorporate synergy into a reference model and lacks information on the dose–response curves for individual drugs [120,121].

Another limitation is the lack of a standard reference model for assessing synergy [107,122]. Additionally, drug–drug interaction analysis in clinical trials is often hindered by practical and ethical constraints that limit the collection of data necessary to properly justify synergy. The choice of reference model must be adapted at each stage of research based on the available data [123].

A striking example of the application of this mathematical model is the study of the interaction effect of TMZ and perifosine. Perifosine’s main mechanism involves blocking the Akt (Protein Kinase B) pathway by preventing Akt from moving to the cell membrane, disrupting its activation, and leading to cancer cell death (apoptosis) and growth inhibition. Thus, these two drugs have two unrelated different mechanisms of action, which is the reason for the use of ZIP [124].

Thus, there are several references models available, but a thorough review of the literature on combination therapy reveals little information about the specific approach used. Despite the large number of reference models, assessing the overall effect of a wide variety of drugs remains difficult for a number of reasons. BBB permeability will vary depending on tumor type, stage, and other factors. Permeability also varies for each drug, and drug distribution itself may vary from the tumor periphery to the core. Nevertheless, we can analyze some experimental studies and make inferences about the methods employed. For example, in a study by [125], the effects of a combination of TMZ, cisplatin, and buthionine sulfoximine were investigated both *in vitro* and *in vivo. In vitro* experiments compared cell death and survival rates, using concentrations of all three compounds that were not effective when used individually. However, a significant effect was observed when they were combined. This suggests a potential indication of the sub-thresholding combination. *In vivo* studies on tumor growth and survival also showed that TMZ had a significant impact, while the other drugs had little effect compared to the control group. When using a combination of all three compounds in therapy, significant effects in inhibiting tumor growth and increasing survival have been observed, which can be theoretically described using the model of the highest efficacy of a single agent. Another example is the use of the combination of TMZ and afatinib [76], where the authors use CompuSyn software to calculate the combination index. This index is calculated according to the Zhou–Talalay method, which uses the Loewe model to take into account the different mechanisms of action of the drugs. Additionally, the use of doxorubicin in combination with TMZ has also been studied, with the authors using Bliss independence as a method for calculating the combination effects [126].

Thus, after reviewing a number of models and some practical application examples, the following recommendations can be made for studying drug interactions: if the substances act on the same target, for example, by blocking the same receptor, enzyme, etc., and have significant dose–response curves, then the Loewe additivity model is used. If the substances act on different targets or independent pathways, then the Bliss independence model is used. If the mechanism is unknown at the study stage, then the nature of the dose–response curves is examined: if the combined effect curve is parallel and shifts left or right, this often indicates the Loewe additivity model, while if the combination curve has a different shape than the individual effects, then the Bliss model is used.

## 6. Conclusions

In conclusion, this review has highlighted the various approaches and multifaceted challenges inherent in glioblastoma therapy, the most difficult disease to treat. The inherent difficulty in treating glioblastoma arises from numerous intricate biochemical pathways that generate crosstalk cascades, thereby diminishing the efficacy of therapeutic agents. Furthermore, challenges related to BBB permeability and the tumor microenvironment restrict the effectiveness of current treatment modalities. Combination therapies can significantly increase patient survival, and the most effective combination of approaches may offer hope for long-term remission. However, to achieve such results, it is necessary to be confident in the significant improvement of the effect of combination approaches compared to monotherapy at the research stage. The analysis of data derived from various drug combinations, such as chemotherapeutic agents integrated with different forms of immunotherapy, can be complex due to several factors. These factors range from an incomplete understanding of the precise mechanisms of drug action to issues pertaining to the generated data (e.g., high levels of dispersion). Given the potential of combination therapies, understanding and demonstrating a ‘synergistic effect’—where the combined therapeutic impact is greater than individual treatments—is critical. This requires a properly selected reference model that considers the mechanism of therapeutic approach at each stage of the study—from *in vitro* to clinical trials. In this review, we delineate the primary therapeutic strategies for glioma that exhibit potential for combinatorial application. Additionally, we present the principal reference models employed for characterizing existing results, which can be applied to individual cases. Appropriate use of reference models and the interpretation of their results will improve the translation of new combination therapeutic approaches for glioblastoma into clinical practice.

## Figures and Tables

**Table 1:** Clinical recommendations for the combination therapy of stage III-IV gliomas (RUSSCO).

Approach for Drug Combination	Drug Combinations	Brief Description (Mechanism of Action)
Increasing the intensity of treatment within one pathway	procarbazine + lomustine	Procarbazine: DNA alkylation and inhibition of macromolecule synthesis; Lomustine: DNA/RNA alkylation leading to crosslinks and apoptosis.
	TMZ + cisplatin	TMZ: alkylates guanine (O6/N7), causing DNA damage and apoptosis; Cisplatin: forms DNA crosslinks that block replication and transcription, leading to apoptosis.
	TMZ + carboplatin	TMZ: see above; Carboplatin: platinum-based drug that forms DNA crosslinks, disrupting DNA function and causing cell death.
Increasing the intensity of chemotherapy targeting separate cellular pathways	procarbazine + lomustine + vincristine	Procarbazine: alkylates DNA and interferes with protein and RNA synthesis; Lomustine: alkylates DNA/RNA (O6-chloroethylguanine) causing crosslinks and cell death; Vincristine: inhibits microtubule formation, blocking mitosis.
	lomustine + vincristine	Lomustine: see above Vincristine: see above
	procarbazine + lomustine	Procarbazine: see above Lomustine: see above
Integration of chemotherapy with immunotherapy	TMZ + bevacizumab	TMZ: see above; Bevacizumab: monoclonal antibody against VEGF that inhibits tumor angiogenesis and can normalize vasculature to improve drug delivery and immune cell access.
	bevacizumab + etoposide	Bevacizumab: see above; Etoposide: inhibits topoisomerase II, causing DNA breaks and apoptosis.
	bevacizumab + irinotecan	Bevacizumab: see above; Irinotecan: topoisomerase I inhibitor that causes single-strand DNA breaks during replication, leading to cell death.
Integration of chemotherapy with drugs against specific mutations	dabrafenib + trametinib	Dabrafenib: BRAF inhibitor that blocks signaling from mutated BRAF V600; Trametinib: MEK inhibitor that blocks downstream MAPK signaling, together suppressing MAPK-driven tumor growth.
	vemurafenib + cobimetinib	Vemurafenib: BRAF inhibitor targeting mutant BRAF V600; Cobimetinib: MEK inhibitor, together reduce MAPK pathway activity and tumor proliferation.

## Data Availability

No new data were created or analyzed in this study. Data sharing is not applicable to this article.
